# Determinants of using children’s mental health research in policymaking: variation by type of research use and phase of policy process

**DOI:** 10.1186/s13012-021-01081-8

**Published:** 2021-01-19

**Authors:** Jonathan Purtle, Katherine L. Nelson, Sarah Mc Cue Horwitz, Mary M. McKay, Kimberly E. Hoagwood

**Affiliations:** 1 Department of Health Management & Policy, Drexel University Dornsife School of Public Health, 3215 Market St, Philadelphia, PA 19104 USA; 2 Department of Child and Adolescent Psychiatry, New York University School of Medicine, 1 Park Avenue, New York, NY 10016 USA; 3 Brown School at Washington University in St. Louis, 1 Brookings Dr, St. Louis, MO 63130 USA

**Keywords:** Policy, Research use in policymaking, Mental health, Children, United States

## Abstract

**Background:**

Research use in policymaking is multi-faceted and has been the focus of extensive study. However, virtually no quantitative studies have examined whether the determinants of research use vary according to the type of research use or phase of policy process. Understanding such variation is important for selecting the targets of implementation strategies that aim to increase the frequency of research use in policymaking.

**Methods:**

A web-based survey of US state agency officials involved with children’s mental health policymaking was conducted between December 2019 and February 2020 (*n* = 224, response rate = 33.7%, 49 states responding (98%), median respondents per state = 4). The dependent variables were composite scores of the frequency of using children’s mental health research in general, specific types of research use (i.e., conceptual, instrumental, tactical, imposed), and during different phases of the policy process (i.e., agenda setting, policy development, policy implementation). The independent variables were four composite scores of determinants of research use: agency leadership for research use, agency barriers to research use, research use skills, and dissemination barriers (e.g., lack of actionable messages/recommendations in research summaries, lack of interaction/collaboration with researchers). Separate multiple linear regression models estimated associations between determinant and frequency of research use scores.

**Results:**

Determinants of research use varied significantly by type of research use and phase of policy process. For example, agency leadership for research use was the only determinant significantly associated with imposed research use (*β* = 0.31, *p* < 0.001). Skills for research use were the only determinant associated with tactical research use (*β* = 0.17, *p* = 0.03) and were only associated with research use in the agenda-setting phase (*β* = 0.16, *p* = 0.04). Dissemination barriers were the most universal determinants of research use, as they were significantly and inversely associated with frequency of conceptual (*β* = −0.21, *p* = 0.01) and instrumental (*β* = −0.22, *p* = 0.01) research use and during all three phases of policy process.

**Conclusions:**

Decisions about the determinants to target with policy-focused implementation strategies—and the strategies that are selected to affect these targets—should reflect the specific types of research use that these strategies aim to influence.

**Supplementary Information:**

The online version contains supplementary material available at 10.1186/s13012-021-01081-8.

Contributions to the literature

- The current study is the first quantitative assessment of how the determinants of research use in policy decision-making vary according to the specific type of research use and phase of policy process when it occurs.
- The determinants of research use vary significantly for different types of research use.
- Dissemination barriers, such as those related to lack of interaction with researchers and lack of actionable messages in summaries of research, are the most universal determinants of children’s mental health research use in policymaking.

## Background

“Evidence-based practice” is central to the enterprise of implementation science, which has roots in clinical health care. However, the concept of evidence-based practice is not directly transferable from clinical to public policy contexts [1]. At best, public policy can be *evidence-informed*, and the *use of research evidence* in policymaking has emerged an accepted indicator of evidence-informed policymaking [2]—with the (albeit untested) [3] assumption that more evidence use results in policy decisions that are more informed by, and aligned with, evidence. Research use in policymaking has been studied for at least half a century, and has long been recognized as a multi-faceted phenomenon. As described by Carol Wiess in the 1970s [4–6], there are a multitude of different types of research use in policymaking. Four specific types have been the focus of extensive study.

First, c*onceptual* (i.e., “enlightenment”) research use relates to that which broadly shapes how a policymaker thinks about an issue. *Instrumental* (i.e., “problem-solving”) research use relates to that in which research directly informs a decision or solves a problem. *Tactical* (i.e., “political” or “symbolic”) research use relates to that in which research is used to persuade someone to see a point of view or support a policy position. Finally, *imposed* research use relates to that which is carried out to satisfy organizational requirements for research use, often with the goal of encouraging instrumental research use [7]. While these specific types of research use have been the focus of extensive qualitative study [8–17], and numerous reviews have documented the determinants of using research in policymaking in general [3, 18–20], surprisingly little quantitative research has assessed variation in the frequency of different types of research use or examined whether the determinants of research use vary according to the type of research use.

### Prior studies

A landmark study by Amara and colleagues was the first to quantify different types of research use in a large sample of policymakers [21, 22]. The study surveyed 833 policymakers of Canadian provincial agencies in 1998 (response rate 35.0%) and characterized the frequency of conceptual, instrumental, and tactical uses of “university research” and identified determinants of research use for these purposes. It was found that all three types of research use occurred fairly frequently and that similar determinants (e.g., agency dedication of resources to support research use, interactions with researchers) were associated with conceptual, instrumental, and tactical use.

Only a few other quantitative studies have examined the prevalence and correlates of policymakers’ uses of research for different purposes. Zardo and Collie examined these issues in a 2012 survey of 372 administrative public health policymakers in Victoria, Australia (response rate = 31.7%) [23, 24]. One analysis of the survey data examined variations in the past-year prevalence of using “academic research” for different purposes and found that a significantly larger proportion of respondents used academic research for conceptual (50.3%) and instrumental (44.8%) than tactical (19.3%) purposes [24]. While this analysis did not assess the determinants of using research for these different purposes, a separate analysis of the survey data used logistic regression to identify factors associated with whether respondents used research evidence for any purpose in the past year [23]. This latter analysis found that factors such as relevance of available research evidence, skills for research use, and organization supports for research use were significantly associated with research use.

Most recently, Williamson and colleagues conducted and scored structured interviews with administrative health policymakers in Sydney, Australia, to quantify the extent of different types of research use in the development of 131 policy documents and to identify associations with determinants of research use [25]. Within this context of policy development, they found that tactical research use (mean = 5.60, 9-point scale) occurred most frequently and that imposed research use (mean = 3.97) occurred least frequently.

### Knowledge gaps: the need for a more nuanced understanding of research use in policymaking

A more nuanced understanding is needed about the dynamics of research use for different purposes in policymaking. This knowledge gap needs to be addressed to develop empirically informed theories about the mechanisms through which implementation strategies could increase the use of research for different purposes in policymaking [26, 27]. In turn, this information is needed to provide an empirical basis to inform the selection of targets and implementation strategies to increase research use in policy processes [26, 28, 29]. There are four specific knowledge gaps that motivate the current study.

First, prior quantitative assessments of different types of research use in policymaking have been limited by simplistic measures—using single-item Likert-scales [21, 22, 25] or dichotomous items [23, 24]. Relatedly, with the exception of Amara’s and colleagues study (conducted more than 20 years ago) [21, 22], the determinants of different types of research use have been measured using single-item dichotomous variables. Second, no studies have focused on how the frequency of research use—in general, or specific type of research use—varies across phases of the policy process. Although the policy process is complex and generally non-linear, there are discrete phases such as deciding which issues to address (i.e., the agenda-setting phase), determining how issues will be addressed and budgeted for (i.e., the policy development phase), and how policies will be rolled out and enforced (i.e., implementation phase) [30].

Third, it is unclear how findings from prior quantitative studies about research use in policymaking—conducted in Canada and Australia—apply to the US context. While surveys have quantified barriers to research use and evidence dissemination preferences among US state agency policymakers [31–35], this work has not examined the frequency of research use. This knowledge gap reflects the fact that policy-focused dissemination and implementation (D&I) research is understudied in the USA. A review of D&I research funded by the National Institutes of Health between 2007 and 2014 found that < 10% of projects explicitly examined policy issues, with most considering policy as a peripheral factor—not the focus of inquiry [36]. An updated review using an identical search strategy identified only 10 additional projects funded between 2015 and 2018 [37]. A review of 22 studies published between 1999 and 2016 that evaluated interventions to increase the capacity for research use in policymaking identified only one study conducted in the USA [26].

Fourth, little prior work about research use in policymaking has focused on mental health, let alone children’s mental health [38]. The topic of children’s mental health in the USA deserves particular attention because rates of depression, anxiety, and suicide among youth having been increasing and could be exacerbated by the stresses of COVID-19 pandemic [39–44]. A review of articles related to evidence-informed mental health policymaking published between 1995 and 2013 identified few rigorous studies, with most studies focusing on the implementation of a specific evidence-based practice at the organizational level—not research use in policymaking more broadly [45]. Only two studies, both qualitative, have explored how research evidence is used in children’s mental health policymaking [13, 14]. A small survey (*n* = 43) of state mental health agency policymakers characterized preferences for receiving, and barriers to using, mental health research but did not assess research use behaviors [32, 33]. Bruns and colleagues used agency and state data (e.g., state per capita income, controlling political party) to identify factors associated with state mental health agencies providing evidence-based treatments for children and adults between 2002 and 2012 [46, 47]. While this work sheds important light on the role of outer-setting context (i.e., the economic, political, and social context that surrounds state agencies) [48] in mental health policymaking, it does not elucidate research use behaviors, or the determinants of these behaviors, among individual policymakers who make policy decisions.

### Current study

The current study addresses these knowledge gaps through a quantitative survey of 224 US state agency officials who are involved with policy decision-making processes related to children’s mental health. The study uses continuous scales to assess and compare different types of children’s mental health research use across phases of the policy process. The study is informed by the SPIRIT Action Framework [49]. The framework is the product of a literature review of research use in policymaking [50], interviews with policymakers [51], and was created and applied in Australian health agencies with the explicit purpose “to guide the development and testing of strategies to increase to use of research in policy” (p. 153) [49].

The following aims of the study are to:

1. Characterize the determinants and frequency of children’s mental health research use in policymaking
2. Assess whether the frequency of children’s mental health research use in policymaking varies according to the type of research use and phase of policy process
3. Identify determinants that are independently associated with the overall frequency of children’s mental health research use in policymaking and assess whether these determinants vary according to the type and phase of research use.

## Methods

### Data

Between December 2019 and February 2020, web-based surveys were conducted of senior-level state mental health agency officials and administrators of grants from the Substance Abuse and Mental Health Service Administration (SAMHSA). State mental health agencies are the government entity responsible for mental health within every state. Officials in these agencies perform functions such as developing and implementing policies and programs and providing and contracting for clinical mental health services [52]. These agencies fund approximately 8,500 providers in the USA annually and who serve a population of 7.3 million [53]. SAMHSA grant administrators—who are typically, but not always, based in state mental health agencies—implement and monitor federal block grants that are a major source of funding for mental health services. For example, the Community Mental Health Services Block Grant program allocated $722 million in fiscal year 2020, $125 million specifically for child mental health services [54].

The survey was created and distributed using Qualtrics, a web-based survey tool. The survey sample frame was created by using contact databases maintained by The National Association of State Mental Health Program Directors’ (NASMHPD) [55] and SAMHSA [56]. Each person in the sample frame was e-mailed eight times with a survey link and telephone follow-up was conducted to ensure that e-mails were received. Respondents were offered a $20 gift card. The survey was sent to 253 SMHA officials with valid e-mail addresses and completed by 129 (response rate = 51.0%), with 63.6% of all state children’s division directors and 57.1% of all state child mental health planners completing the survey. Four-hundred-twelve SAMHSA grant administrators with valid e-mail addresses were sent the survey and it was completed by 95 (response rate = 23.1%). At least one survey was completed in 49 states (respondents per state range = 1, 10; median = 4; 98% of states responding) and the total aggregate sample size was 224 (aggregate response rate = 33.7%).

All survey items explicitly pertained to decision-making and use of research evidence related to *children’s mental health*, not mental health broadly. Because it was likely that some respondents would not have knowledge about or experience with decision-making related to children’s mental health, a “not applicable to me in my work” response option was provided for each question. The order of the survey items in each domain of questions was randomized to reduce the risk of order-effect bias [45]. Using a definition aligned with that of the US Commission on Evidence-Based Policymaking [57], “research” was defined in the survey as “information produced by using reliable data and systematic methods—such as findings reported in peer-reviewed publications or from analyses of local, state, or national data.” This definition appeared above each question that pertained to research use. The survey instrument was piloted with seven former state mental health officials and experts in children’s mental health policy and two telephone-based focus group sessions were conducted to receive feedback on the survey instrument to ensure clarity and comprehensiveness.

### Dependent variables

The primary dependent variable was the overall frequency of children’s mental health research use, operationalized as a score (range = 12, 60) that was the sum of responses to 12 items that assessed the frequency of four types of research use (i.e., conceptual, instrumental, tactical, imposed) across three phases of the policy process (i.e., agenda setting, policy development, policy implementation). The score had strong internal consistency (Cronbach’s alpha = 0.90). The score was created using items adapted from the Seeking, Engaging with and Evaluating Research (SEER) instrument [58], which was developed to operationalize aspects of the SPIRIT Action Framework. The original SEER instrument assesses these four types of research use but was modified to assess them across separate phases of the policy process and on 5-point Likert scales instead of yes/no items. For each phase of the policymaking process, respondents were separately asked to indicate the frequency (1 = “very rarely,” 5 = “very frequently”) with which they engaged in each of the four types of children's mental health research use. When answering these questions, respondents were instructed to respond in reference to their “general experience over the past three years.” An overall frequency of children’s mental health research use score was not calculated for a respondent if they selected “not applicable to me in my work” in response to any of the items used to calculate the score.

Consistent with feedback obtained when piloting the survey, we did not explicitly name the types of research use or phases of policy process. Rather, we defined each in lay terms that were consistent with definitions used in the SEER instrument [58]. The exact wording used for each type of research use and phase of policy process is included in Supplemental Table 1.

The secondary dependent variables were the frequency of *each type* of children’s mental health research use (summing the scores for each type across all three phases of policy process) and frequency of children’s mental health research use during *each phase* of policy process (summing the scores for all four types of research use within each phase). The scores that quantified each type of research use had a range of 3 to 15 and high internal consistency (Cronbach’s alpha ranging from 0.79 to 0.95) and the scores that quantified research during each phase of the policy process had a range of 4 to 16 and also had high internal consistency Cronbach’s alpha ranging from 0.72 to 0.82). These composite scores were not calculated for a respondent if they selected “not applicable to me in my work” in response to any of the items used to calculate the scores.

### Independent variables

The independent variables were composite scores assessing four domains of determinants of using children’s mental health research in policymaking. The selection of these variables was informed by the SPIRIT Action Framework [49], reviews of barriers to evidence-informed policymaking [3, 18–20], and prior research related to evidence use in mental health policymaking in the USA [13, 32, 36, 59, 60]. The exact wording of all questions is in Supplemental Table 1.

*Skills for research* use were assessed by a score that was the sum of responses to three, five-point Likert scale items adapted from the SEER instrument [58] (Cronbach’s alpha = 0.86). *Agency leadership for research* use was assessed by a score that was the sum of responses to two, five-point Likert scale items also adapted from the SEER instrument (Cronbach’s alpha = 0.75, *r* = 0.61). *Agency barriers to research use* were assessed by a score that was the sum of responses to three, five-point Likert scale items adapted from prior assessments of barriers to using research evidence in US state policy contexts (Cronbach’s alpha = 0.53) [33]. *Research dissemination barriers* were assessed by a score that was the sum of responses to four, five-point Likert scale items that have also been used in prior assessments of barriers to research use in US state policy contexts (Cronbach’s alpha = 0.71) [33].

### Covariates

Ordinal variables characterized respondents’ highest level of education and the number of years they had worked at their agency. The US state of respondents’ agency was also included as a covariate.

### Analysis

Means were calculated for all composite scores as well as individual items. To aid interpretation, descriptive statistics were also generated with each individual item treated as a dichotomous variable in which responses of 1-3 were coded as “no” and 4-5 were coded as “yes.” Paired sample *t* tests compared the mean composite frequency of research use scores for each type of research use and research use during each phase of the policy process. Pearson product-moment bivariate correlations and partial correlations assessed relationships between the composite scores for each domain of determinant of research use. These correlations were depicted in accordance with recommendations for Gaussian graphical models [61].

Multiple linear regression was used to estimate associations between the determinants and frequency of children’s mental health research use. Assessment of multi-collinearity revealed that the variance inflation factor was between 1.0 and 2.0 for all independent variables in all models, indicating the absence of multi-collinearity [62]. Assessment of the normality of the data revealed that the agency leadership for research composite score (skewness = −0.47) and agency barriers to research composite score (skewness = −0.89) were negatively skewed at a threshold ≥ 0.20. Thus, these scores were log transformed when entered into the models. To make the interpretation regression coefficients consistent, the skills for research use and dissemination barrier composite scores were also log transformed when entered in the models. Frequency of research use composite scores was skewed at a threshold ≥ 0.20 and was thus also log transformed. All models adjusted for respondent highest level of education, years working at their agency, and state.

First, the overall frequency of children’s mental health research use score served as the dependent variable in a set of models that sequentially added determinant of research use composite scores as independent variables. Then, each of the four frequency of different types of research use composite scores served as the dependent variable in a separate model that adjusted for all determinants of research use composite scores and covariates. The same analysis procedure was conducted with each of the three phases of policy process composite research use scores as the dependent variable in a separate model. We compared the size and statistical significance of beta coefficients for each determinant of research use across these models to assess variation in the determinants of different types of research use and research use during different phases of the policy process.

Because many US states and the federal government have implemented requirements for imposed research use in policymaking [57, 63], we conducted two exploratory analyses related to the frequency of imposed research use. First, we classified survey respondents according to whether their state had a law requiring the use of research evidence in mental health policymaking, defined as such by a 2017 Pew-MacArthur Foundation report [63]. We then used independent sample *t* tests to compare the mean composite frequency of imposed research use score between respondents who did and did not this law in their state. Second, because imposed research can be perceived as an antecedent to instrumental research use [7], we calculated Pearson product-moment correlations between the frequency of imposed and instrumental research use.

## Results

### Respondent characteristics and determinants of children’s mental health research use in policymaking

Table 1 shows the characteristics of respondents and the prevalence of possible determinants of using children’s mental health research in policymaking. The mean *skills for research use* composite score was 11.23 (SD = 2.91; 15-point scale). Seventy percent of respondents indicated that they had confidence in their ability to find children’s mental health research, while 59.6% had confidence in their ability to evaluate the quality of this research. The mean *agency leaderships for research use* composite score was 7.24 (SD = 1.89; 10-point scale). Three-quarters (76.5%) of respondents expressed that leadership in their agency believed it was important to use children’s mental health research, but only 42.1% believed that their agency dedicated resources to promote the use of this research. The mean *agency barriers to research use* composite score was 9.66 (SD = 2.59; 15-point scale) with 58.3% of respondents indicating that limited agency resources (e.g., budget deficits) was a barrier. The mean *dissemination barriers to research use* composite score was 11.86 (SD = 3.28; 20-point scale) with lack of actionable messages/recommendations in summaries of research (43.1%) and lack of interaction or collaboration with researchers 37.2% most frequently identified as barriers in this domain.

**Table 1:** Characteristics of survey respondents and determinants of using children’s mental health research in policy decision-making, State Agency Officials, Winter 2019-2020, *N* = 224

	*N*	*Mean*	*SD*	*%*^a^
Demographics				
Highest level of education				
Some college, college degree	34			15.4
Master’s degree	130			58.8
Doctoral degree	57			25.8
Years working at agency				
≤ 2	46			20.8
3-5	31			14.0
6-9	43			19.5
≥ 10	101			45.7
Determinants of research use				
Skills for research use (range = 3-15)	196	11.2	2.91	
Confidence in ability to find children’s MH research	200	3.90	1.11	70.5
Confidence in the ability to interpret the results of children’s MH research	200	3.81	1.06	68.5
Confidence in the ability to evaluate the quality of children’s MH research	198	3.54	1.12	59.6
Agency leadership for research use (range = 2-10)	221	7.24	1.89	
Agency leadership believes it is important to use children’s MH research	221	4.05	0.92	76.5
The agency dedicates resources to promote the use of children’s MH research	221	3.18	1.17	42.1
Agency barriers to research use (range = 3-15)	187	9.66	2.59	
Limited agency resources (e.g., budget deficits)	199	3.72	1.11	58.3
Lack of time to use research	191	3.36	1.20	47.1
Unable to access to research articles	197	2.62	1.30	28.4
Research dissemination barriers (range = 4-20)	174	11.9	3.28	
Lack of interaction or collaboration with researchers	183	3.06	1.18	37.2
Lack of actionable messages/recommendations in summaries of research	195	3.16	1.15	43.1
Questions that researchers ask are not relevant to decisions	184	2.90	1.10	29.3
Unclear presentation/communication of research findings	195	2.74	1.05	21.5

*MH* mental health

^a^Determinant of research use variables dichotomized as 4-5 of 5-point scale

**Fig. 1:**
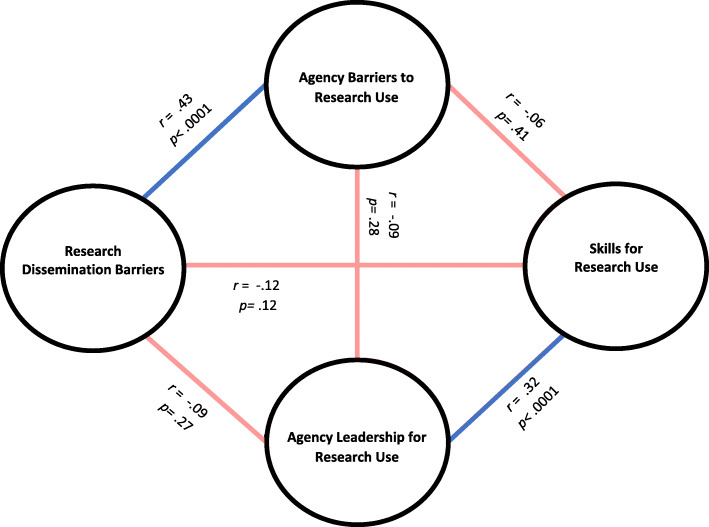
Correlations between determinants of using children’s mental health research in policy decision-making, State Agency Officials, Winter 2019-2020, *N* = 224. Note. Dark blue lines indicate positive partial correlations, *p* ≤ 0.01; light red lines indicate negative partial correlations with *p* > 0.01, but negative non-partial bivariate correlations *p* ≤ 0.01. Correlation coefficients and *p* values are only displayed for partial correlations that adjusted for the other two domains of determinants of research use that were not being correlated

Figure 1 depicts correlations between the composite scores for all determinants of children’s mental health research use. There were weak (*r* ≤ 0.3) but significant (*p* ≤ 0.006) bivariate correlations between each determinant score, with the exception of a moderately strong correlation between dissemination barriers and agency barriers (*r* = 0.63, *p* < 0.001). In partial correlations that adjusted for the other domains of determinants, there were significant positive correlations between research dissemination barriers and agency barriers for research use (*r* = 0.43, *p* < 0.0001) and agency leadership for research use and skills for research use (*r* = 0.32, *p* < 0.0001).

### Frequency of using children’s mental health research in policymaking

Table 2 presents summary statistics about the frequency of using children’s mental health research in policymaking. The overall frequency of children’s mental health research use was 44.26 (SD = 8.23; 60-point scale). There were no significant differences in the composite frequency scores between conceptual (mean = 12.11; SD = 2.32), instrumental (mean = 12.01; SD = 2.26), and tactical (mean = 12.07; SD = 2.40) research use (*p* ≥ 0.52 for all comparisons). However, the imposed research use composite score (mean = 8.02; SD = 3.57) was significantly lower (*p* < 0.0001 for all comparisons). There were no significant differences in the composite frequency of research use scores across phases of agenda setting (mean = 14.84; SD = 2.86), policy development (mean = 14.70; SD = 3.17), or policy implementation (mean = 14.49; SD = 3.34) (*p* ≥ 0.08 for all comparisons).

**Table 2:** Frequency of using children’s mental health research in policy decision-making, State Agency Officials, Winter 2019-2020, *N* = 224

	*N*	*Mean*	*SD*	*%*^a^
Overall frequency of children’s MH research use (range = 12-60)	171	44.26	8.23	
Composite frequency of different types of research use				
Conceptual	178	12.11	2.32	
Instrumental	181	12.01	2.26	
Tactical	184	12.07	2.40	
Imposed	176	8.02	3.57	
Composite frequency of research use during different phases of the policy process				
Agenda	181	14.84	2.86	
Development	178	14.70	3.17	
Implementation	182	14.49	3.34	
Frequency of different types of child MH research use across phases of the policy process				
Conceptual, agenda	191	4.03	0.91	75.4
Conceptual, development	187	4.01	0.98	74.9
Conceptual, implementation	192	3.94	0.98	74.5
Instrumental, agenda	191	4.04	0.88	74.9
Instrumental, development	188	3.96	0.89	76.6
Instrumental, implementation	192	3.93	0.93	74.5
Tactical, agenda	193	4.10	0.84	76.7
Tactical, development	190	3.99	0.98	73.2
Tactical, implementation	193	3.92	1.01	69.9
Imposed, agenda	186	2.66	1.25	24.7
Imposed, development	179	2.67	1.23	25.7
Imposed, implementation	183	2.70	1.27	26.2

*MH* mental health

^a^Frequency of research use variables dichotomized as 4-5 of 5-point scale

**Fig. 2:**
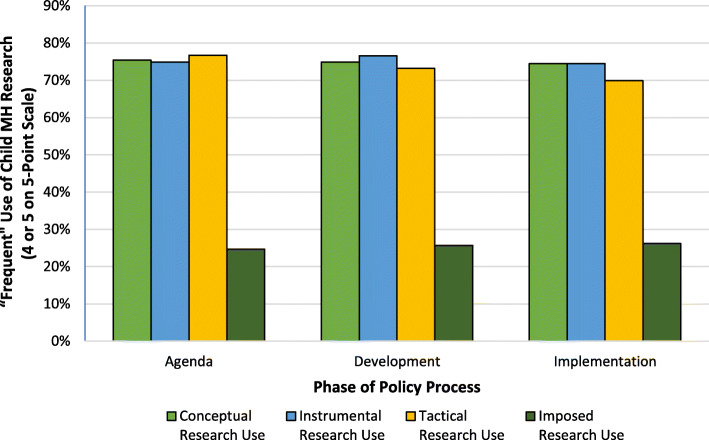
Frequency of different types of children’s mental health research use across different phases of the policy process, State Agency Officials, Winter 2019-2020, *N* = 224. MH, mental health

When children’s mental health research use variables were dichotomized and analyzed as individual items, the majority of respondents frequently (i.e., 4 or 5 on the 5-point scale) engaged in conceptual, instrumental, and tactical research use—ranging from 76.7% for tactical use in the agenda phase to 69.9% for tactical use in the implementation phase. The proportion of respondents who frequently engaged in imposed research use ranged from 24.7% in the agenda-setting phase to 26.2% in the policy implementation phase. To illustrate how the use of research did not vary across phases of the policy process, Fig. 2 depicts the proportion of respondents who frequently engaged in each type of research use during each phase of the policy process. Supplemental Figure 1 contains this figure with means displayed instead of percentages and shows a nearly identical pattern.

### Associations between determinants of research use and frequency of research use: variation by type of research use and phase of policy process

Table 3 shows adjusted associations between each domain of determinant of children’s mental health research use and overall frequency of research use score. In the fully adjusted model, agency leadership for children’s mental health research use (*β* = 0.25, *p* < 0.01) and dissemination barriers (*β* = −0.21, *p* < 0.01) were significantly associated with the frequency of research use. The interpretation of these coefficients is that, after adjustment, a 1% increase in agency leadership is associated with a 0.25% increase in the frequency of research use, and a 1% reduction in dissemination barriers is associated with a 0.21% increase in research use.

**Table 3:** Adjusted associations between determinants of using children’s mental health research and overall frequency of children’s mental health research use, State Agency Officials, Winter 2019-2020, *N* = 224

	*β*	*p*	*B*	B Lower (95% CI)	B Upper (95% CI)
State	0.14	0.06	0.00	0.00	0.00
Highest level of education	0.03	0.66	0.01	−0.02	0.03
Years working at agency	0.08	0.31	0.00	0.00	0.01
Agency leadership for research use	0.25***	< 0.001	0.17	0.06	0.28
Skills for research use	0.12	0.14	0.09	−0.03	0.22
Agency barriers to research use	−0.06	0.52	−0.04	−0.15	0.08
Research dissemination barriers	−0.21*	0.02	−0.14	−0.25	−0.02
*Model fit statistics*					
Adjusted *R*^2^	0.18				
*F*	5.57				
*F* (Sig)	< 0.001				

*p ≤ 0.05

**p ≤ 0.01

***p ≤ 0.001

Table 4 shows the results of fully adjusted models in which composite frequency of research use scores for each of the four types of research use served as the dependent variable in a separate model. A different combination of determinants of research use and covariates were significantly associated with the frequency of research use for each purpose. In terms of *conceptual* use of children’s mental health research, the numbers of years that a respondent had been working at their agency (*β* = 0.16, *p* = 0.03), skills for research use (*β* = 0.18, *p* = 0.02), and dissemination barriers (*β* = −0.22, *p* = 0.01) were significantly associated with the frequency of use. For *instrumental* research use, only dissemination barriers (*β* = −0.22, *p* = 0.01) were significantly associated with the frequency use. In regard to *tactical* research use, the only determinant significantly associated with the frequency of use was skills for research use (*β* = 0.17, *p* = 0.03). Finally, in terms of *imposed* research use, the only determinant significantly associated with the frequency of use was agency leadership for research use (*β* = 0.31, *p* < 0.001). The interpretation is that, after adjustment, a 1% increase in agency leadership for research use is associated with a 0.31% increase in the frequency of imposed research use. The magnitude of the association between agency leadership for research use and imposed research use was twice as large as the association between this determinant and the frequency of any other type of research use.

**Table 4:** Adjusted associations between determinants of using children’s mental health research and composite frequency of different types of research use, State Health Agency Officials, Winter 2019-2020, *N* = 224

	Model 1: Conceptual research use					Model 2: Instrumental research use					Model 3: Tactical research use					Model 4: Imposed research use				
	*β*	*p*	*B*	B Lower 95% CI	B Upper 95% CI	*β*	*p*	*B*	B Lower 95% CI	B Upper 95% CI	*β*	*p*	*B*	B Lower 95% CI	B Upper 95% CI	*β*	*p*	B	B Lower 95% CI	B Upper 95% CI
State	0.08	0.30	< 0.001	< 0.001	< 0.001	0.11	0.15	< 0.001	< 0.001	< 0.001	0.13	0.08	< 0.001	< 0.001	< 0.001	0.13	0.10	< 0.001	< 0.001	< 0.001
Highest level of education	0.03	0.67	0.01	-0.02	0.03	0.03	0.68	0.01	-0.02	0.03	0.14	0.07	0.02	<0.001	0.05	−0.13	0.10	−0.05	−0.10	0.01
Years working at agency	0.16*	0.04	0.01	< 0.01	0.01	0.04	0.61	< 0.001	−0.01	0.01	0.10	0.20	0.001	0.001	0.01	0.04	0.63	0.01	−0.01	0.02
Agency leadership for research use	0.12	0.12	0.09	-0.02	0.21	0.15	0.06	0.11	−0.01	0.23	0.09	0.25	0.07	−0.05	0.20	0.31**	< 0.001	0.54	0.27	0.82
Skills for research use	0.18*	0.02	0.16	0.02	0.29	0.09	0.28	0.08	−0.06	0.21	0.17*	0.04	0.15	0.01	0.30	-0.04	0.58	-0.09	−0.41	0.23
Agency barriers to research use	−0.07	0.43	−0.05	−0.17	0.07	−0.08	0.34	−0.06	−0.18	0.06	−0.03	0.76	−0.02	−0.15	0.11	−0.07	0.43	-0.12	−0.41	0.18
Research dissemination barriers	−0.22**	0.01	−0.15	−0.27	−0.03	−0.22**	0.01	−0.15	−0.28	−0.03	−0.14	0.12	−0.10	−0.23	0.03	−0.06	0.48	−0.10	−0.40	0.19
*Model fit statistics*																				
*Adjusted R*^*2*^	0.17					0.11					0.09					0.122				
*F*	5.50					3.79					3.27					4.05				
*F* (Sig)	< 0.001					0.001					0.003					< 0.001				

*p ≤ 0.05

**p ≤ 0.013 (Bonferroni corrected *p* value threshold for four model comparisons in the table)

There was also variation in the determinants that were associated with the frequency of research use within each phase of the policy process (Table 5). During the *agenda-setting phase*, state (*β* = 0.17, *p* = 0.02), skills for research use (*β* = 0.16, *p* = 0.04), and dissemination barriers (*β* = −0.20, *p* = 0.02) were significantly associated with frequency of research use. In both the *policy development phase* and the *implementation phase*, agency leadership for research use (*β* = 0.27 and *β* = −0.21, respectively, both *p* < 0.01) and dissemination barriers (*β* = 0.18 and *β* = −0.24, respectively, both *p* ≤ 0.05) were significantly associated with the frequency of research use.

**Table 5:** Adjusted associations between determinants of using children’s mental health research and composite frequency of research use across different phases of policy process, State Health Agency Officials, Winter 2019-2020, *N* = 224

	Model 1: Agenda phase research use					Model 2: Development phase research use					Model 3: Implementation phase research use				
	β	*p*	B	B Lower 95% CI	B Upper 95% CI	β	*p*	B	B Lower 95% CI	B Upper 95% CI	β	*p*	B	B Upper 95% CI	B Lower 95% CI
State	0.17*	0.02	< 0.001	< 0.001	< 0.001	0.10	0.21	< 0.001	< 0.001	< 0.001	0.08	0.28	< 0.001	< 0.001	< 0.001
Highest level of education	0.03	0.71	0.001	-0.02	0.03	0.06	0.44	0.01	-0.02	0.04	0.02	0.84	< 0.001	-0.02	0.03
Years working at agency	0.09	0.24	< 0.001	< 0.001	0.01	−0.01	0.89	< 0.001	-0.01	0.01	0.10	0.22	0.01	0.00	0.01
Agency leadership for research use	0.12	0.13	0.08	−0.02	0.19	0.28**	< 0.001	0.24	0.10	0.38	0.21**	0.01	0.18	0.04	0.32
Skills for research use	0.16*	0.05	0.13	0.00	0.25	0.04	0.64	0.04	−0.13	0.20	0.10	0.20	0.10	−0.06	0.26
Agency barriers to research use	−0.12	0.18	−0.08	−0.19	0.04	−0.04	0.67	−0.03	−0.18	0.12	0.01	0.91	0.01	−0.14	0.15
Research dissemination barriers	−0.20*	0.03	−0.13	−0.24	−0.02	−0.18*	0.05	−0.15	−0.30	0.00	−0.24**	0.01	−0.20	−0.35	−0.05
*Model fit statistics*															
*Adjusted R*^*2*^	0.16					0.11					0.13				
*F*	5.12					3.71					4.40				
*F* (Sig)	< 0.001					0.001					< 0.001				

*p ≤ 0.05

**p ≤ 0.016 (Bonferroni corrected *p* value threshold for three model comparisons in the table)

### Exploratory analysis related to the frequency of imposed research use

Forty-three percent of survey respondents worked in a state that had a law requiring the use of research evidence in mental health policymaking. There was no significant difference in the frequency of imposed research use between respondents who did and did not have this law in their state (7.88 vs. 8.13, *p* = 0.47). There was a significant correlation (*r* = 0.42, *p* < 0.0001) between the frequency of imposed and instrumental research use. However, the strength of these correlations was not substantially different than those of correlations between imposed and conceptual (*r* = 0.35, *p* < 0.0001) or imposed and tactical (*r* = 0.38, *p* < 0.0001) research use.

## Discussion

We aimed to characterize the determinants and frequency of children’s mental health research use in US state policymaking and understand how these constructs vary by type of research use and phase of policy process. We find that the most common barriers to using children’s mental health services research relate to limited agency resources, agencies not dedicating resources to promote research use, and insufficient time to use research. We also find that research evidence is regularly used for a diversity of purposes throughout the policy process, with the vast majority of respondents frequently using research conceptually, instrumentally, and tactically across all three phases of the policy process. With the exception of imposed research use, which was found to occur least frequently, we observe that the frequency of research use does not vary according to the type of research use or phase of policy process. However, we find that the determinants of research use do vary by type of research use and phase of policy process. Of note, a different combination of determinants of research use and covariates were significantly associated with the frequency of each type of research use. This finding raises questions about why there is variation in the determinants of different types of research use and how this information can be used to inform the design of policy-focused implementation strategies.

Many of the variations observed in the determinants of research use intuitively make sense. For example, skills for research use, but no other domains of determinants, were significantly associated with the frequency of tactical research use. It is logical that the policymakers who are most adept at finding, interpreting, and evaluating the quality research evidence have the most ability to strategically use research findings for persuasive purposes [8]. It also makes sense that agency leadership for research use was the only domain of determinant associated with imposed research use. This is because agency leaders—and the leadership to whom they are accountable (e.g., governors, state legislators)—have the authority to require that their staff use research evidence in policymaking. The magnitude of this association was also two times larger than the association between agency leadership for research use and the frequency of conceptual, instrument, and tactical research use.

Consistent with prior research [7, 25], we found that the mean frequency of imposed research use score was significantly lower than that of other types of research use. Our exploratory analysis found no significant association between state laws that require the use of research evidence in mental health policymaking and the frequency of imposed research use. Relatedly, our exploratory correlational analyses do not provide strong support for the notion that imposed research is an antecedent to instrumental research use. While future research is needed to examine these exploratory findings in greater depth, the findings suggest that research use in policymaking is not something that can be simply mandated by state laws or other imposed requirements.

The finding that the determinants of research use vary for different types of research use suggests that decisions about the determinants to target with policy-focused implementation strategies should reflect the specific types of research use that these strategies intend to affect. This raises an important question for the field: which types of research use should be prioritized? Are all types of research use equally important, or are some more critical to evidence-informed policymaking than others? For example, should instrumental research use be prioritized because it is most proximal to a concrete policy decision? Should tactical research be a lower priority because it can be [8, 16, 64], although is not always, used to promote political gain and policies that are misaligned with evidence? While the literature provides little guidance on these questions, an implication of our findings is that this may be an important area for future debate and consensus building.

An alternative approach, which negates the need to focus on some types of research use and not others, is to target determinants that increase the overall frequency of research use in policymaking. In this case, our findings suggest that implementation strategies that increase agency leadership for research use and reduce dissemination barriers would be most effective and efficient [65], given that these two domains of determinants were significantly associated with the overall frequency of children’s mental health research use. In terms of strategies to increase agency leadership for the use of children’s mental health use, approaches could be adapted from those that have demonstrated success at improving leadership for evidence-based practice at organization and clinical levels [66]. Examples of such approaches include the Leadership and Organizational Change for Implementation model [67], the iLead model [68], and the Ottawa Model of Implementation Leadership (O-MILe) [69, 70]. Future research could explore how these models might be adapted to focus on state policymakers and research use in decision-making as opposed to organization leaders and specific EBPs.

In terms of addressing dissemination barriers, guidance exists in recent reviews that have synthesized information about strategies to enhance the policy impact of health research [33, 71–77]. Many of these strategies relate to either the packaging of research evidence (e.g., enhancing the relevance and presentation of evidence summaries) or fostering collaboration between researchers and policymakers. For example, one strategy to improve the packaging of evidence for policymakers is to use local, as opposed to national, data to characterize a problem, and highlight policies to address it. A 2017 survey found that the vast majority (93%) of state mental health agency officials identified “relevance to residents in my state” as a “very important” feature of mental health research [32]. Field experiments conducted in the USA and the UK have found that policymakers’ engagement with research evidence can be increased by tailoring evidence summaries so that the geographic level of the data presented corresponds with the population they serve [78, 79].

In terms of fostering collaboration between researchers and policymakers, many models have demonstrated success. In the USA, Family Impact Seminar model (state level) [80–83] and the Research-to-Policy Collaboration model (federal level) [84] serve as examples. The William T. Grant Foundation has also synthesized guidance about approaches in this domain [85]. Outside of the USA, the SPIRIT model in Australia included a component intended to increase interactions between researchers and policymakers [86], and intermediary organizations in Canada are supported by government funds to help facilitate these connections [87]. While none of these models focus on children’s mental health in US state contexts, they offer guidance to inform the selection of implementation strategies that build on the findings of our study.

### Limitations

The results of our study should be considered within the context of its scope and limitations. The study was broadly focused on the uses of research in state children’s mental health policymaking and not research evidence related to a specific mental health issue, intervention, or policy. Relatedly, the survey used a broad definition of research evidence and did not assess the uses of different types of evidence (e.g., testimony of families, agency reports). Prior qualitative research suggests that different types of evidence might be used in different ways [12, 13].

The aggregate response rate of 33.7% is considered good for a sample of policymakers [88] and consistent with prior surveys of administrative policymakers about uses or research evidence [21–24]. However, it is possible that survey respondents are not fully representative of all children’s mental health policymakers in US states, although 98% of all states had at least one respondent. It should be noted, however, the response rates were high for key stakeholder groups in our sample (e.g., 63.6% of all state children’s division directors and 57.1% for state child mental health planners). Our analyses did not focus on differences in research use between different types of decision-makers (e.g., agency directors vs. children’s division directors, SAMHSA grant administrators vs. non-SAMHSA grant administrators) and such comparisons are an area for future research.

Given that a relatively small number of respondents were from each state (median = 4), it is possible that the study was underpowered to fully capture state effects (although this was not an aim of the study). There would be value to future research which links survey data with administrative data about state agency context—similar to the data used by Bruns and colleagues in their analysis of outer-context factors associated with state mental health agency provision of evidence-based treatments [46, 47].

## Conclusion

The frequency of children’s mental health research use in state agency policymaking does not vary according to the phase of policy process or type of research use, with the exception of imposed research use which occurs least frequently. Importantly, however, there is significant variation in the determinants of different types of research use. This suggests that decisions about the determinants to target with policy-focused implementation strategies—and the strategies that are selected to affect these targets—should be aligned with the specific types of research use that these strategies aim to influence.

## Supplementary Information

**Additional file 1: Supplemental Figure 1.** Frequency of Different Types of Children’s Mental Health Research Use across Different Phases of the Policy Process, State Agency Officials, Winter 2019-2020, N=224.

**Additional file 2: Supplemental Table 1.** Wording of Survey Items.

## Data Availability

The datasets used and/or analyzed during the current study are available from the corresponding author on reasonable request.
