# Supplementary material for: Determinants of using children’s mental health research in policymaking: variation by type of research use and phase of policy process
Source: Implement Sci. 2021 Jan 19;16:13. doi: 10.1186/s13012-021-01081-8 (PMC7815190; doi:10.1186/s13012-021-01081-8)
Supplement: Supplementary file 1 — Additional file 1: Supplemental Figure 1. Frequency of Different Types of Children’s Mental Health Research Use across Different Phases of the Policy Process, State Agency Officials, Winter 2019-2020, N=224. [file 13012_2021_1081_MOESM1_ESM.docx]

**Supplemental Figure 1: Frequency of Different Types of Children’s Mental Health Research Use across Different Phases of the Policy Process, State Agency Officials, Winter 2019-2020, N=224**

MH= mental health
