# Supplementary material for: Determinants of using children’s mental health research in policymaking: variation by type of research use and phase of policy process
Source: Implement Sci. 2021 Jan 19;16:13. doi: 10.1186/s13012-021-01081-8 (PMC7815190; doi:10.1186/s13012-021-01081-8)
Supplement: Supplementary file 2 — Additional file 2: Supplemental Table 1. Wording of Survey Items. [file 13012_2021_1081_MOESM2_ESM.docx]

**Supplemental Table 1: Wording of Survey Items**

| **Frequency of Research Use Items** |  |
| --- | --- |
| Conceptual research use | “Use research to help you understand how to think about an issue”  1= Very rarely, 5= Very frequently |
| Instrumental research use | “Use research to decide about content or direction of a policy or program”  1= Very rarely, 5= Very frequently |
| Tactical research use | “Use research to persuade others to a point of view or course of action”  1= Very rarely, 5= Very frequently |
| Imposed research use | “Use research because your organization required you to do so”  1= Very rarely, 5= Very frequently |
| Agenda setting phase | “When preliminary discussions are taking place about whether to develop or change a policy or program to address a children’s mental health issue”  1= Very rarely, 5= Very frequently |
| Policy development phase | “When decisions are being made about the details of a children’s mental health policy or program, or setting the annual budget related to children’s mental health”  1= Very rarely, 5= Very frequently |
| Policy implementation phase | “When a children’s mental health policy or program is being implemented and rolled-out in real-world setting”  1= Very rarely, 5= Very frequently |
| **Determinants of Research Use Items** |  |
| Skills for research | “Please indicate the extent to which you feel confident that you have the knowledge and skills to...   - Find children’s mental health research to inform policy or program development - Evaluate the quality of children’s mental health research - Interpret the results of children’s mental health research”   1= Not very confident, 5= Very confident |
| Agency leadership for to research use | “To what extent do you agree with the following statements about your agency?   - Agency leadership believes it is important to use children’s mental health research in policy or program development - The agency dedicates resources to promote the use of children’s mental health research”   1= Strongly disagree, 5= Strongly agree |
| Agency barriers to research | “To what extent do you perceive each issue as a barrier to using children's mental health research in your work?   - Lack of time to use research - Unable to access to research articles - Limited agency resources (e.g., budget deficits)”   1= Not a barrier, 5= Major barrier |
| Research dissemination barriers | “To what extent do you perceive each issue as a barrier to using children's mental health research in your work?   - The questions that researchers ask are not relevant to the decisions I make - Unclear presentation/ communication of research findings - Lack of interaction or collaboration with researchers - Lack of actionable messages and recommendations in summaries of research”   1= Not a barrier, 5= Major barrier |
